# Collaborative weighting in federated graph neural networks for disease classification with the human-in-the-loop

**DOI:** 10.1038/s41598-024-72748-7

**Published:** 2024-09-19

**Authors:** Christian Hausleitner, Heimo Mueller, Andreas Holzinger, Bastian Pfeifer

**Affiliations:** 1 Institute for Medical Informatics, Statistics and Documentation, Medical University Graz, 8036 Graz, Austria; 2 Human-Centered AI Lab, Institute of Forest Engineering, Department of Forest and Soil Sciences, University of Natural Resources and Life Sciences Vienna, 1190 Vienna, Austria; 3 Alberta Machine Intelligence Institute, Edmonton, T6G 2R3 Canada

**Keywords:** Computational platforms and environments, Machine learning, Software

## Abstract

The authors introduce a novel framework that integrates federated learning with Graph Neural Networks (GNNs) to classify diseases, incorporating Human-in-the-Loop methodologies. This advanced framework innovatively employs collaborative voting mechanisms on subgraphs within a Protein-Protein Interaction (PPI) network, situated in a federated ensemble-based deep learning context. This methodological approach marks a significant stride in the development of explainable and privacy-aware Artificial Intelligence, significantly contributing to the progression of personalized digital medicine in a responsible and transparent manner.

## Introduction

**Graph Neural Networks (GNNs)** play a crucial role in biology, especially in biomedicine, by effectively capturing complex relationships within biological systems represented as graphs^1^. Their ability to model interconnected networks is valuable for integrating diverse biological data types, aiding in the understanding of molecular mechanisms, disease pathways, and potential therapeutic targets and they facilitate the discovery of new structural classes, particularly in heterogeneous information networks^2–4^, highly relevant e.g. in drug repositioning to discover new indicators of drugs^5^. The capacity of graph neural networks to represent interlinked networks proves beneficial for amalgamating various types of biological data. This assists in comprehending molecular mechanisms, disease pathways, and potential therapeutic targets. Additionally, they play a crucial role in identifying new structural categories, such as in the development of antibiotics^6^, or to predict core gene candidates for complex diseases^7^, to give only a few examples.

Network Medicine leverages network science to elucidate disease mechanisms, employing a variety of analytical methods to construct molecular networks such as protein-protein interactions and gene regulatory networks, and applies these to Omics Big Data for advancements in diagnosis, prognosis, and treatment of complex diseases^8–11^. GNNs have shown great promise in predicting interactions, identifying disease subtypes, and leveraging large-scale, multi-modal data in biomedicine^12–15^.

At the same time, **Federated Learning (FL)** enhances collaborative model training by enabling decentralized learning without sharing any critical data. However, FL can not only support diagnostic data protection, but also has other advantages, namely improving the reproducibility and reliability of AI models outside the domain and potentially optimising results^16,17^. In combination with GNNs this holds great promise for advancing personalized medicine, disease understanding, and drug discovery on a large scale^18^. Due to the fact that most existing medical data is not fully utilized because it is hoarded in data silos due to privacy concerns^19^, federated learning may offer a solution for the future of digital health^20^.

Incorporating the **Human-in-the-Loop (HITL)** in federated learning introduces a critical dimension of human oversight and interaction, enhancing the model’s interpretability, reliability, and adaptability. The benefit of bringing the human into the algorithmic loop has been shown in a wide range of studies^21–24^. This integration is particularly pivotal in domains requiring nuanced decision-making, such as healthcare and personalized services, where human expertise and ethical considerations play a significant role. Integrating a human expert into federated learning with GNNs adds an extra layer of refinement and interpretability.

In this work we delineate a novel approach for disease classification employing federated Graph Neural Networks (GNNs) augmented with collaborative weighting, which innovatively incorporates the expertise of human domain professional knowledge directly into the deep learning process. The foundational work of Pfeifer et al.^25,26^ utilized a Protein-Protein Interaction (PPI) network as the underlying structure for the GNNs. In this model, each patient is represented as a unique PPI network, with nodes augmented by specific molecular markers derived from gene expression and DNA methylation data. This modeling approach results in a graph classification problem and can be applied to any binary outcome class, such as distinguishing between healthy patients and those with a specific disease.

In a significant advancement we employed explainable AI (xAI) methods to dissect the PPI knowledge graph into several subgraphs^25,26^. These subgraphs then serve as the foundation for constructing an ensemble classifier. The predictive model functions through a majority voting mechanism based on insights gleaned from these subgraphs, thereby enhancing the robustness and accuracy of disease classification. This enables the experts to gain deeper insights into the underlying explanatory factors and thus strengthen conceptual understanding and trust^27^.

Here, we introduce an advanced collaborative and interactive framework that seamlessly integrates human expertise into the aforementioned algorithmic process, a paradigm shift in the realm of machine learning. This integration enables a more nuanced exploration of the ensemble classifier, providing human experts with the capability to adjust the predictive model by experimenting with varying weights assigned to the ensemble subgraphs.

Crucially, this framework empowers experts to delve into the significance of specific proteins and genes within the Protein-Protein Interaction (PPI) knowledge graph. Leveraging the principles of explainable AI, it facilitates a deeper understanding of molecular interactions and their implications in disease pathology. This approach is particularly transformative as it allows the infusion of external expert knowledge into the network, knowledge that may not be inherently present in the training dataset. Such an inclusion of expert insight, especially regarding the nuances of gene functions and interactions, can significantly enhance the model’s predictive accuracy and reliability. This synergy between algorithmic robustness and human expertise stands to make substantial contributions to the field of digital medicine generally and biomedical research specifically.

Our framework has been operationalized as an application within the FeatureCloud platform^28^. This platform is architecturally composed of a global frontend, backend, and a localized controller. It employs Docker, a pivotal technology, to segregate local computational components from sensitive data infrastructures, thus maintaining data integrity. FeatureCloud streamlines the complexities inherent in distributed systems, offering a robust and scalable infrastructure for conducting Federated Learning analyses across multiple institutions. Moreover, it supports efficient algorithm implementation. A significant aspect of FeatureCloud is its integrated artificial intelligence store, which serves as a nexus for the communal exchange of federated algorithms, enhancing collaborative scientific efforts. Central to FeatureCloud’s design is its commitment to data privacy. The platform incorporates privacy-enhancing technologies that safeguard locally shared models, ensuring compliance with the European General Data Protection Regulation (GDPR)^29^, thereby fortifying trust in its data management practices.

## Materials and methods

### The FeatureCloud framework

Our approach has been seamlessly integrated into the FeatureCloud platform^28^. FeatureCloud is a Python framework that enables developers to create multi participant workflows, without having to deal with any backend implementation or hosting services. It provides a server that coordinates data distribution between the clients, which is hosted by themselves. The framework is intended to be used with a clients/coordinator structure. Both use the same code, but the framework provides data to separate them at runtime.

The workflows are separated into states, for code encapsulation and better progress overview. Each state should represent a workflow part. It also features a command line interface. Each FeatureCloud app is a Docker container, which needs to use the FeatureCloud package and expose the port of the control web server, so the app implementation can communicate with the FeatureCloud Controller. A second port can be exposed to host a web application as UI. Other than that, the developer is free to implement everything else he needs inside the docker container. FeatureCloud also provides a convenient way to show a UI to the user. It will be rendered either as an embedded iFrame in their workflow overview or can be detached to run in a separate Browser tab. It has to be noted, that the web application is rerouted through the FeatureCloud controller, which puts some limitations on the way it can be rendered. Especially with XSS policies. For end users, they only have to install docker and the *featurecloud* pip package, which starts the Docker container with the controller, and participate in a workflow. The rest is handled by the app implementation and the FeatureCloud web UI.

### FeatureCloud app for interactive and federated deep learning

**Figure 1:**
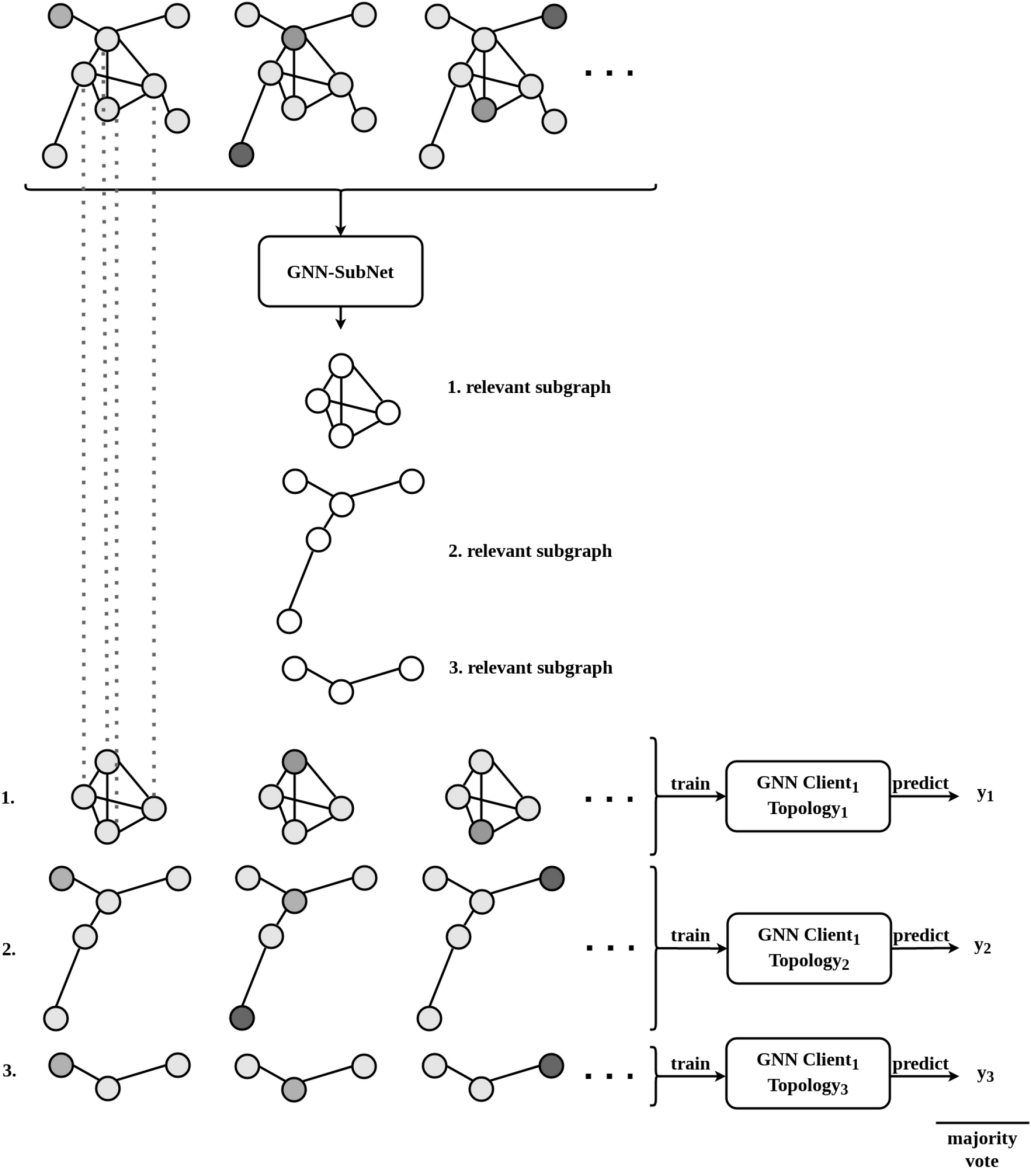
Illustration of the implemented ensemble Graph Neural Network. Explainable AI identifies the relevant subgraphs that are used to train a GNN classifier, with each trained model becoming a member of an ensemble^30^.

**Figure 2:**
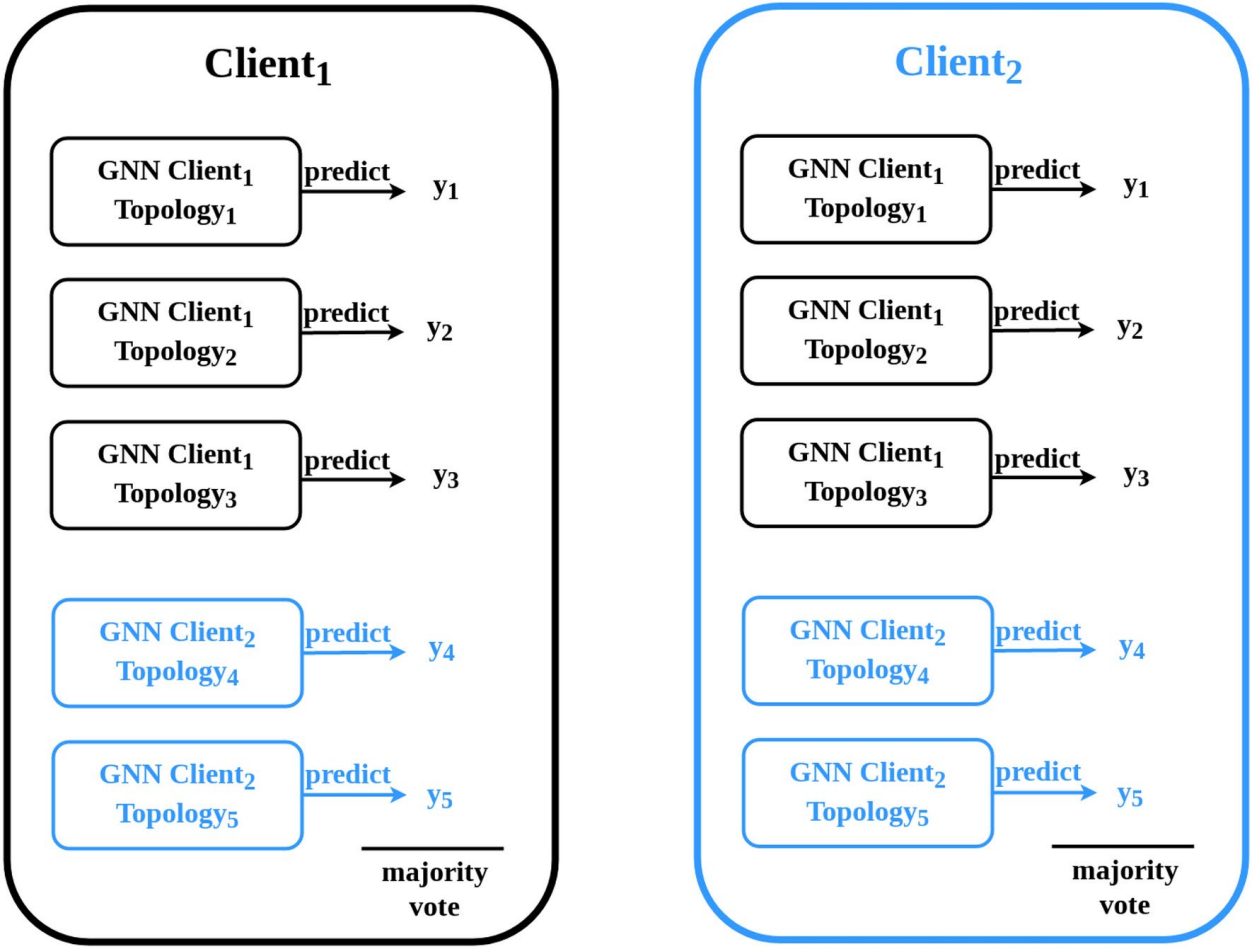
Construction strategy of the federated ensemble classifier. The topology of each *local* ensemble member is shared and concatenated to a *global* federated ensemble classifier^30^.

The implemented algorithm is based on previous work [cite]. With the herein presented work, however, we have made it fully actionable for federated computing incorporating human-in-the-loop principles. Essentially, the algorithm expects gene expression data in tabular form, plus a Protein-Protein interaction network specifying the biological dependencies between the studied markers. Each patient is represented as a PPI network and the nodes are enriched using the patient-specific gene expression values. This modelling process, detailed in Fig. 1 , results in a graph classification problem fully processable by GNNs. Not only it can be processed; due to the vast amount of explainable AI algorithms for GNNs, it is also possible to verify parts of the PPI network which were most relevant for the predictions. Based on these verified subnetworks an ensemble classifier is constructed. As can be seen from Fig. 1 each ensemble member consists of a different PPI subnetwork and topology. These are shared with the other clients and are concatenated to a global federated classifier without sharing any patient-specific data (see Fig. 2). Breaking down the model into smaller components enhances the domain expert’s understanding of the model behavior. Within our developed GUI the domain-expert can inspect these networks, track the performance and the relevant parts of the nodes and edges relevant for prediction. Furthermore, the weighted majority vote of the obtained ensemble can now be influenced by the domain-expert in an collaborative and federated manner. The core idea is that multiple experts can contribute their knowledge to the federated ensemble classifier without the necessity to share any patient data. The domain-expert is able to inspect the different subnetworks and can assign weights to it based on his knowledge e.g about the biological relevance of the associated proteins. Ultimately, the expert shares the weights with the participants of the federated collaborative workflow. The specified weights effect the majority vote and will finally converge to a collaborative consensus voting. The interactive GUI is presented and discussed in the results and discussion section.

**Figure 3:**
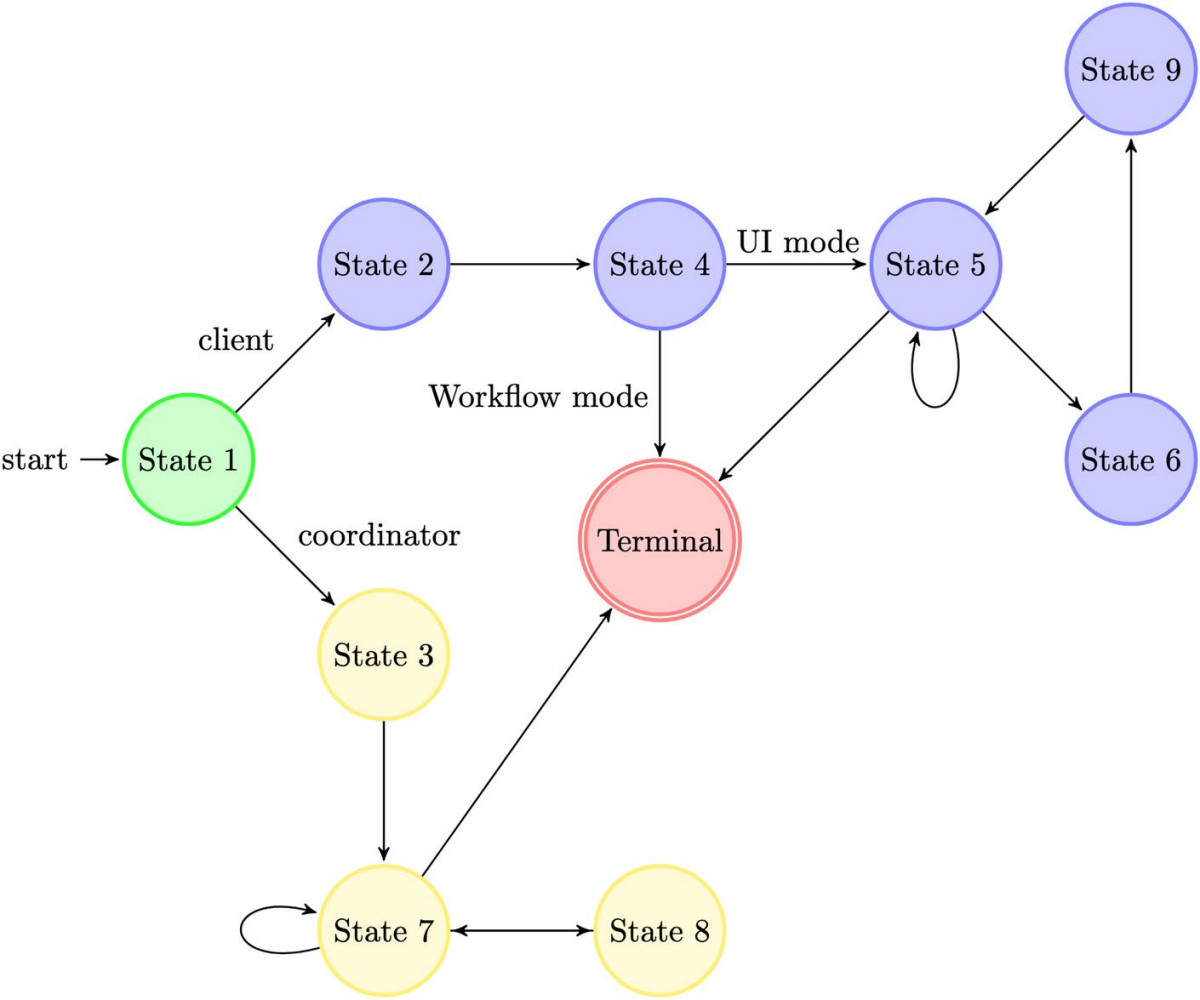
The states of our workflow. Green: client and coordinator states, Blue: client only states, Yellow: coordinator only states.

The system is implemented as a state machine with 9 states (8 working states + Terminal state). The possible transitions are shown in Fig. 3 and explained in the next part. The design of a state machine was recommended by the FeatureCloud framework.

#### State 1—Initial (Coordinator/Client)

When the machine is the coordinator, the number of clients is stored, and it transitions to the “Global model aggregation state”. If the machine is a participant, the config file is loaded from the input directory. The file paths of the input data and the UI Mode flag are stored on the client side.

#### State 2—Local training (Client only)

The stored paths of the input files are loaded again and the data is copied to the output directory. The files passed to the algorithm to initialize the GNN. The loaded input data gets split up into a train-, test- and validation dataset. The GNN is trained on the training data. To start the global aggregation, the ensemble classifiers are detached from the input data and send to the coordinator. The detachment ensures, that only the trained network is distributed, without any user data. After successful distribution of the classifiers, the performance of the model is tested on the validation and test set and stored.

#### State 3—Global model aggregation (Coordinator only)

The number of clients is loaded, and the coordinator waits until all clients have sent their data. When completed, all client models are combined to a aggregated global model. This is done by appending the trained ensemble classifiers from all clients to the global model^26^. Before transitioning to the next state, the coordinator broadcasts the global model to all clients.

#### State 4—Waiting for global model (Client only)

The client waits the model from the coordinator. After receiving, the model is stored as global model on the client. The global model is tested on the local validation and test set data of the client. If the UI mode flag is not set, the workflow is complete. The performance of the local and global model is stored to a file in the output directory, the coordinator is notified that the client is finished and the client terminates. If the UI mode flag is set, the client transitions to the “Web controlled” state.

#### State 5—Web controlled (Client only)

The purpose of this state is to keep the app running and ready for any user action. The user can interact with the API for the following actions:

- Terminate the client
- Change the weights of the local model and get the new performance statistics
- Weight global model—Change the weights of the global model and get the new performance statistics
- Send the weights of the global model to the coordinator, to get an average of the weighting from all clients. This creates a transition to the “Distribute weights” state.

#### State 6—Distribute weights (Client only)

The client sends the weights to the coordinator. The weights consist of an ordered list of values, which are defaulted to 1 in case no change is made. To ensure a client only sends one weight configuration, the sending action is blocked in the UI until the aggregation is complete.

#### State 7—Waiting for clients to finish (Coordinator only)

The coordinator waits for the data from each client. This can either be a notification that the client terminates or a list of weights for the global weight aggregation. In the case of the terminal notification, the coordinator stores the decreased number of active clients. If no clients are active anymore, the coordinator terminates. If some clients send a list of weights, the weight configurations are stored and the workflow transitions to state 8.

#### State 8—Global weight aggregation (Coordinator only)

The previously stored weights are loaded, and a safety check is executed. In case a weight configuration has missing values, it is truncated with weight 1. After this, the weights are averaged to get a single list of values, where each value is the average of all values at the same index from all the input lists. Finally, the aggregated weight configuration is broadcasted to all clients.

#### State 9—Waiting for global weights (Client only)

The client waits until it received the weights from the coordinator. They are stored as the global federated weight configuration and the client transitions back to state 5.

#### Wrapping up

The states describe two state machines: The coordinator as a single instance and the clients, which can be multiple instances. All clients can be individual, but they have to synchronize for certain actions like weight aggregation or model aggregation (which is handled by the coordinator).

## Results and discussions

In our pursuit of advancing user-centric methodologies in artificial intelligence, we have developed a sophisticated Graphical User Interface (GUI) for the interactive analysis and manipulation of graph-based deep learning models. Recognizing the growing significance of human-centered design approaches in scenarios where a human-in-the-loop plays a pivotal role in the efficacy of learning algorithms, our GUI aligns with contemporary research trends^31,32^. The criticality of user interfaces in the realm of explainable AI is increasingly acknowledged, necessitating focused academic inquiry^33^.

To ensure maximal intuitiveness and user engagement, the GUI’s design is rooted in Google’s Material Design principles. This design philosophy draws inspiration from the tangible world, particularly in how materials such as paper and ink interact with light and cast shadows, thereby creating a user experience that feels natural and grounded in reality. By emulating these physical properties, the GUI offers an accessible and visually coherent interface, essential for facilitating effective user interaction with complex graph-based deep learning systems.

**Figure 4:**
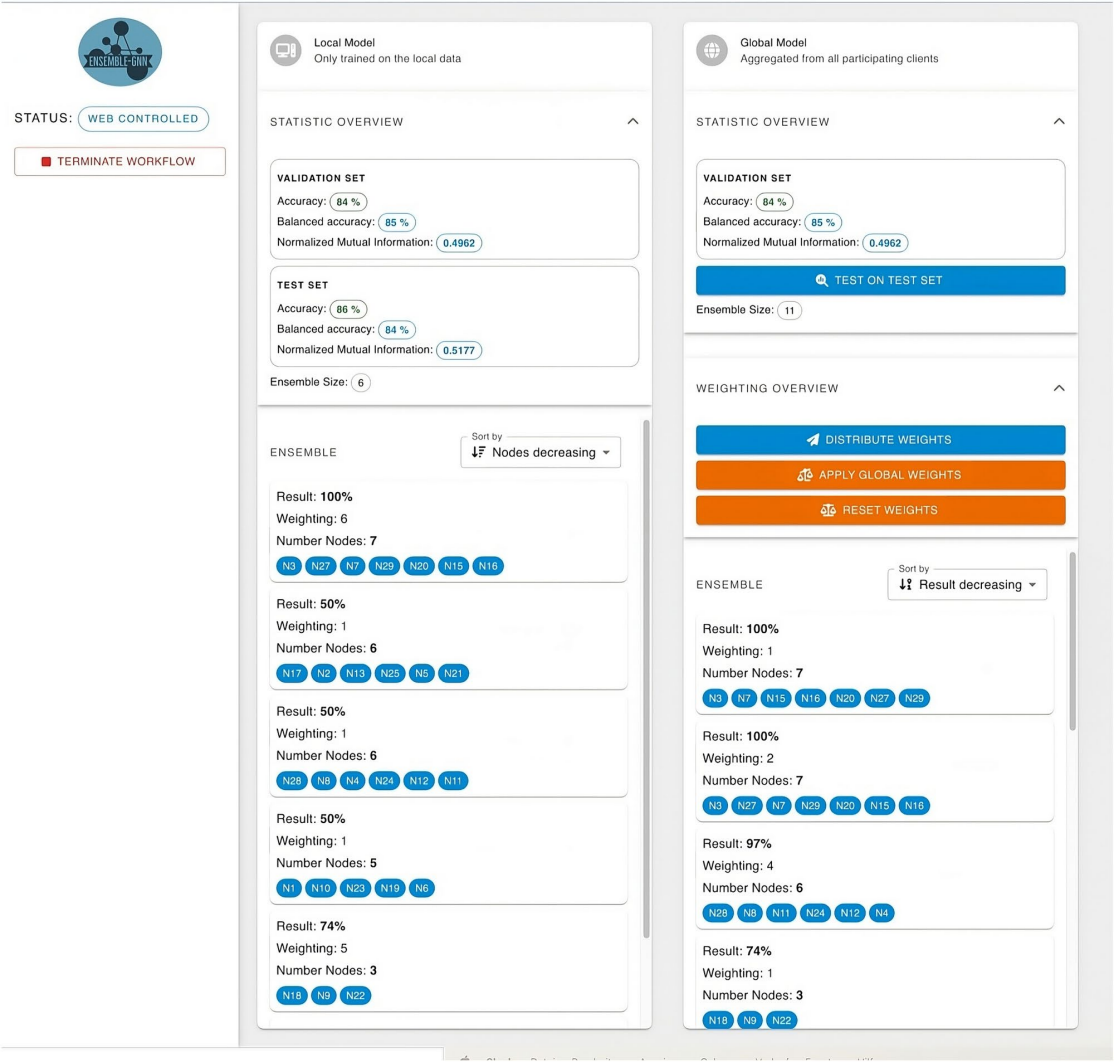
The main view of our UI. The status bar on the left and the model containers for the local and the global model on the right side are displayed. The gene names of a specific subgraphs and the performance of the associated classifiers; for the ensemble classifier as well as for each member of the ensemble.

**Figure 5:**
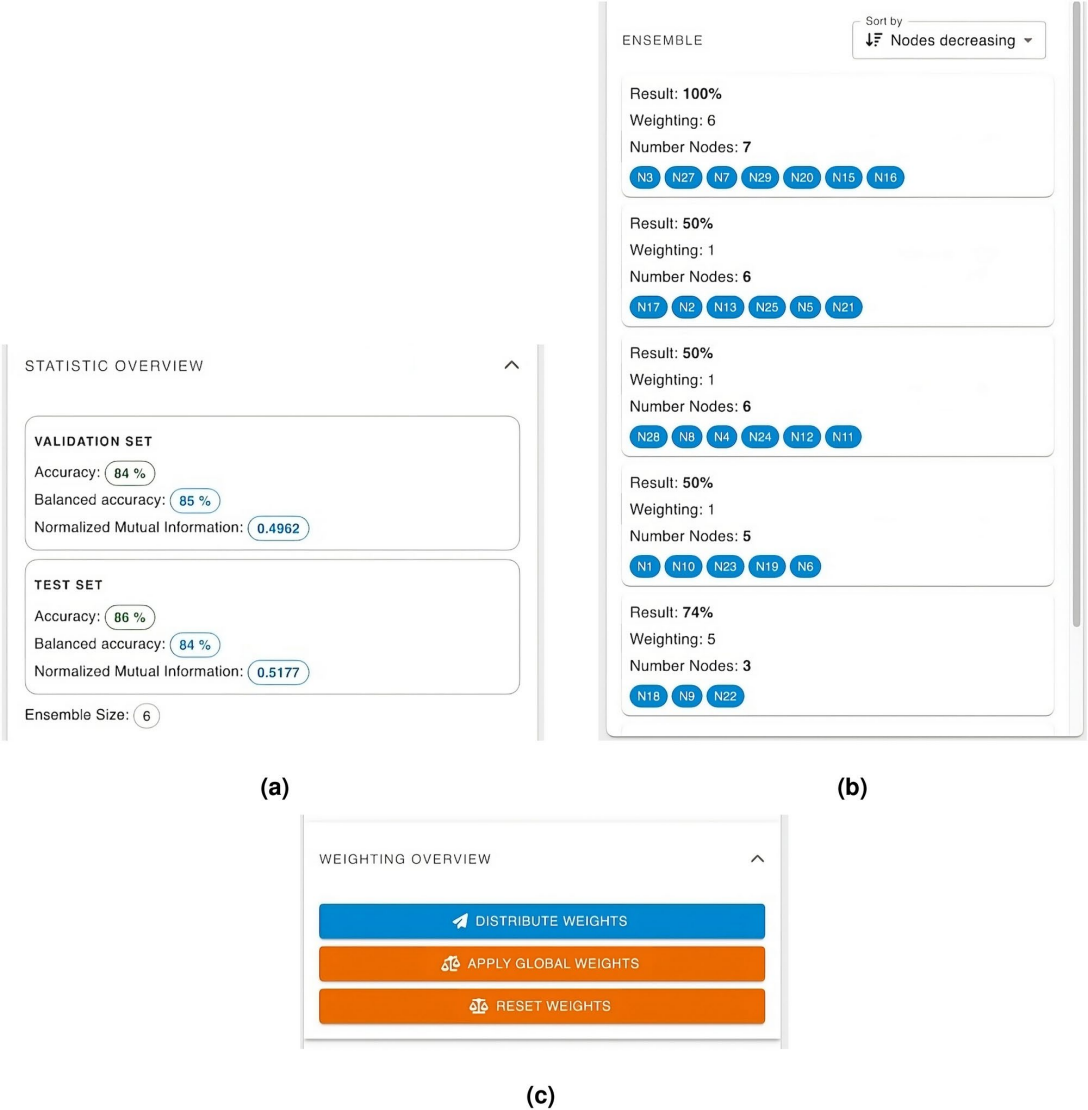
Data visualization of a single model. (**a**) Shows the performance after testing on the validation and test set. (**b**) A list of ensemble classifiers with their individual performance and a list of their genes are displayed. In (**c**) we see the collaborative actions which can be done on the global model, which are handled by states 6–9 (see Fig. 3).

The UI can be separated into three main components: The **sidebar**, the **model container** and the **model popup**. The **sidebar** is the fixed container displayed on the left side of Fig. 4. It displays the current state, the loading status of the training process and a button to terminate the client. It also displays error messages and additional information banners. The **model container** is used for the local and global model. The statistics from both the validation and test sets are presented here, providing a clear comparison and insight into the performance metrics across these distinct data subsets (see Figs. 5 and 4).

Every change of the weights is send to the server, which re-calculates the performance of the updated model based on the validation set.

Below the statistics section, the global model features the weighting overview. Here the user can reset all weights to 1 (neutral), distribute his engineered weights to the global weight distribution process, and/or apply the global aggregated weights, if available. Also, a sortable list of the ensemble classifiers including their metadata is shown (see Figs. 5 and 4). By clicking, the **model popup** opens. The model popup has additional three tabs: The **graph rendering**, the **data table** and the **weighting menu**.

**Figure 6:**
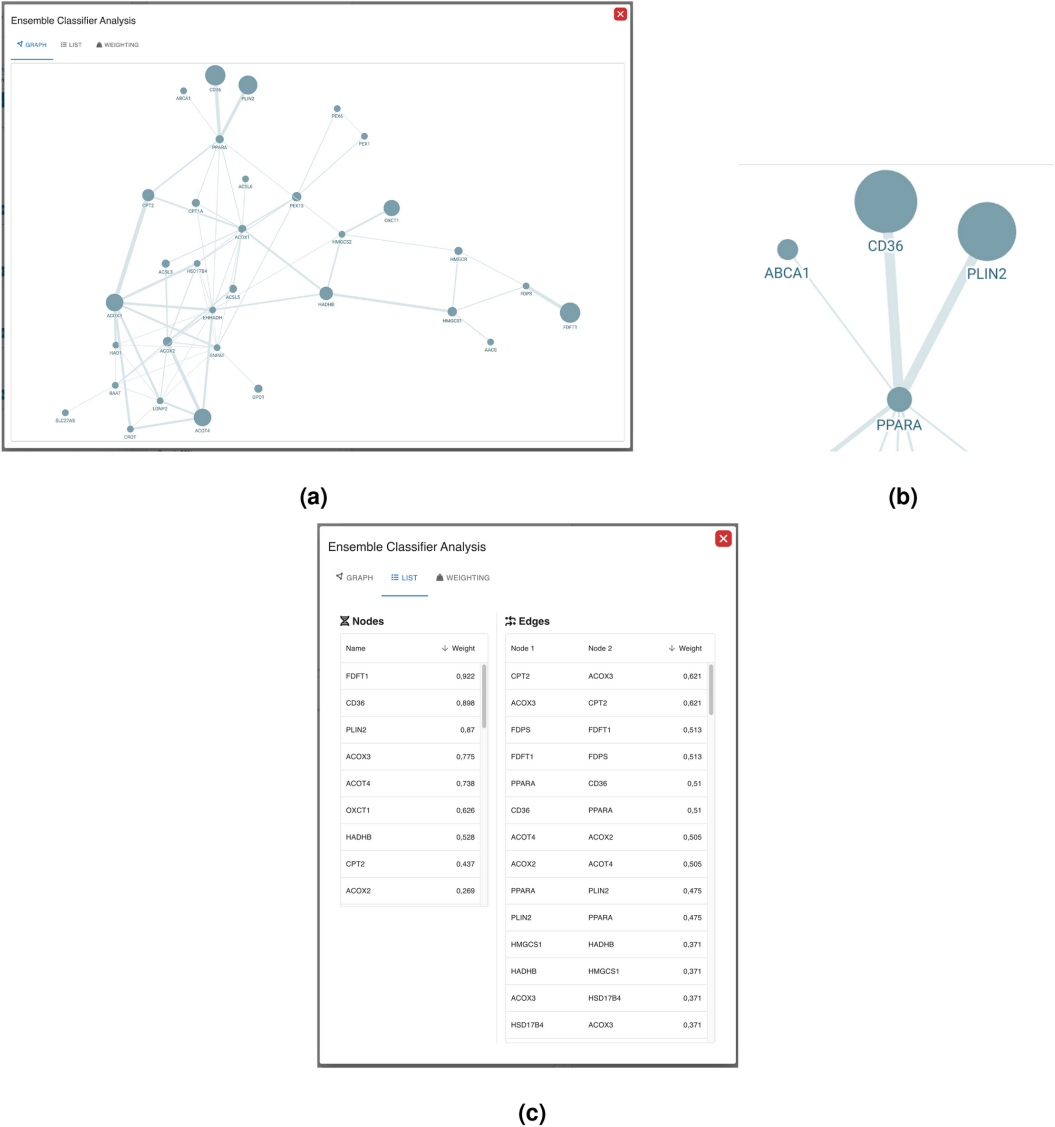
(**a**) The topology of the associated knowledge graph, that can be zoomed and panned. (**b**) The relevant components of the classification are reflected by the thickness of the edges and the size of the nodes using explainable AI techniques; with greater importance being denoted by increased thickness and larger node size. (**c**) The edge and node/gene importance values from (**a**) are displayed as tables.

**Figure 7:**
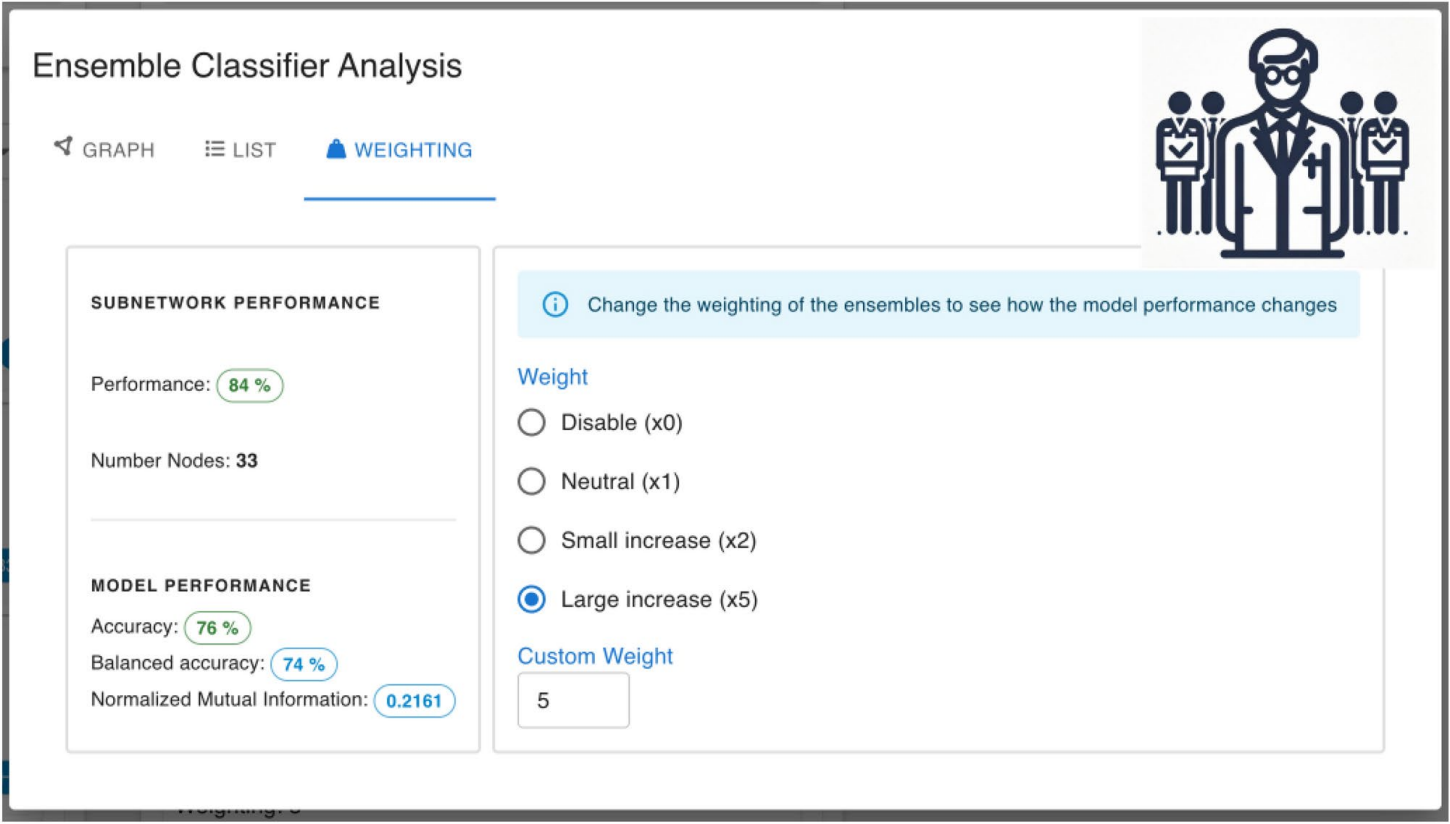
Collaborative ensemble weighting. The ensemble weights as part of the majority vote can be modified and shared with the other participants. The resulting performance of the federated model including the aggregated weights are calculated on an independent validation set after each weight change.

Figure 6 shows the **graph rendering** and the **data table** sections, enabling the user to inspect the data from an ensemble subgraph. The **weighting menu** features the weighting of the ensemble classifier (see Fig. 7). The weights can be changed by the user and the resulting performance of the classifier is displayed.

## Data Availability

The code is available via https://github.com/pievos101/fc-ensemble-gnn The App is available via the FeatureCloud App-Store: https://featurecloud.ai/app/ensemble-gnn.
